# Prevalence and Antibiotics Susceptibility Pattern of* Salmonella* and* Shigella* Species among Diarrheal Patients Attending Nekemte Referral Hospital, Oromia, Ethiopia

**DOI:** 10.1155/2018/9214689

**Published:** 2018-01-24

**Authors:** Alemayehu Terfassa, Mulissa Jida

**Affiliations:** ^1^Department of Biology, College of Natural and Computational Sciences, Wollega University, Nekemte, Ethiopia; ^2^Environmental Biotechnology Directorate, Ethiopian Biotechnology Institute, Addis Ababa, Ethiopia

## Abstract

The main objective of this study was determining the prevalence and antibiotics resistance pattern of* Salmonella* and* Shigella* sp. from diarrheal patients attending Nekemte Referral Hospital. A total of 422 patients were included in the study and their sociodemographic and clinical information was collected using questionnaire. Stool samples of the patients were collected and processed following standard bacteriological protocols. Presumptive colonies of* Salmonella* and* Shigella* species were identified and subcultured to their respective genera by standard biochemical tests. Antibiotics susceptibility of the isolates was tested using disk diffusion assay. The prevalences of* Salmonella and Shigella* sp. among the patients were 7.1% and 2.1%, respectively. The antimicrobial susceptibility test results of the isolates showed that they are highly resistant to amoxicillin (30 *μ*g). In contrast, the isolates showed relatively lower resistance level to ceftriaxone (30 *μ*g), nalidixic acid (30 *μ*g), norfloxacin (10 *μ*g), and ciprofloxacin (5 *μ*g). This study revealed 9.2% prevalence of* Salmonella* and* Shigella* sp. which were resistant to commonly prescribed antibiotics. Thus, intervention measures such as health education, provision of safe drinking water, improvement of waste disposal systems, and surveillance of antibiotics susceptibility of the pathogens should be done regularly.

## 1. Introduction

Acute gastroenteritis is one of the leading causes of illness and death in infants, children, immune-compromised, and aged individuals throughout the world. Of the enteric pathogens* Salmonella* and* Shigella* species are of particular concerns as causes of enteric fevers, food poisoning, and gastroenteritis [1, 2]. They are transferred from person to person usually by asymptomatic carriers and via contaminated food and water. They are Gram-negative rods which commonly inhabit intestinal tracts of humans and many animals [3–5]. Currently,* Salmonella *and* Shigella species *are progressively becoming resistant to the commonly used antimicrobial agents [6–9]. The emergence of antibiotic resistance among their species is serious problem in developing countries.

Diseases caused by* Salmonella* and* Shigella* species are common public health problems in many parts of the world, including Ethiopia [10–13]. Studies conducted in different cities of Ethiopia indicated that they are widespread among diarrheal patients [8, 14–16]. Moreover, their increased resistance to commonly used antimicrobials has also been noted [13–16]. On the contrary, there is no information on their prevalence and antimicrobial resistance patterns in Nekemte. Therefore, this study was aimed at determining prevalence and antimicrobial resistance patterns of* Salmonella* and* Shigella* species among diarrheal patients attending Nekemte Referral Hospital.

## 2. Materials and Methods

### 2.1. Study Area, Design and Population

This study was conducted in Nekemte Referral Hospital from October 2015 to February 2016. Nekemte is located in Western parts of Oromia regional state at geographical location of 9°46′N latitude and 36°31′E longitude, with elevation ranging from 1,960 m to 2,170 m a.s.l. The study populations were all patients with acute diarrhea attending Nekemte Referral Hospital. Patients who were under antibiotics treatment in the last 14 days of sampling period were excluded from the study.

A cross-sectional study design was carried out to determine the prevalence and antimicrobial resistance pattern of* Salmonella* and* Shigella* species among diarrheal patients attending Nekemte Referral Hospital. Pretested questionnaire was used to gather information concerning sociodemographic data, clinical characteristics, and hygiene practice of the patients.

### 2.2. Sampling and Sample Size Determination

The study participants were selected via systematic random sampling technique from OPD of the hospital. A minimum sample size of 384 was calculated using single population proportion formula, assuming 95% confidence interval, 50% prevalence, and 5% marginal error; as a result, 422 patients were included in the study [17].

### 2.3. Stool Sample Collection and Handling

Stool specimens were collected in sterile container and transported to Microbiology Laboratory, Oromia Regional Health Laboratory, Nekemte, within 2 hrs of collection. All plates and test tubes were incubated at 37°C for 24 hrs.

### 2.4. Isolation of* Salmonella* and* Shigella* Species

About 1 g of the stool sample was transferred to Selenite F broth and incubated at 37°C for 24 hrs. Simultaneously, a loop full of stool sample suspension was streaked on SSA and XLDA and then incubated at 37°C for 24 hrs. Furthermore, culture negative specimens on SSA and XLDA media were subcultured from Selenite F broth enrichment broth to SSA and XLDA plates to improve recovery of the isolates. Growth of* Salmonella* and* Shigella* species were detected by their typical colonies characteristic on SSA and on XLDA [18]. All isolates were purified by repeated subculturing, streaked on nutrient agar slant and preserved in refrigerator set at 4°C until further analysis.

### 2.5. Morphological and Biochemical Characterization of the Isolates

#### 2.5.1. Morphological Characterization

Purified single colony from SSA/XLDA was taken with loop, spread on the center of clean slide, and subject to Gram staining. Finally, the stained smear was observed under microscope (oil immersion objective) and then morphology, cell arrangement, and Gram reaction of the isolates were determined.

#### 2.5.2. Motility Test

To determine whether the tested organism was motile or not, motility media were inoculated with a straight inoculating needle through a single stab about 1-2 cm down into the medium and incubated at 37°C for 24 hrs. Based on their appearance on motility medium the test organisms were categorized as motile and nonmotile.

#### 2.5.3. Biochemical Characterization of the Isolates

Colonies with typical characteristics of* Salmonella *and* Shigella* species from primary media were further cultured on Kligler Iron Agar (KIA) medium by stabbing the butt and streaking the slant and then incubated at 37°C for 24 hrs to determine their glucose and lactose fermentation abilities and production of hydrogen sulfide [19]. The KIA tubes were examined for specific growth and appearance of* Salmonella* and* Shigella *species. Colonies of the isolates from primary media were further examined by stabbing the butt and streaking on surface of the slant of Lysine Iron Agar (LIA) and incubating at 37°C for 24 hrs. The growth of positive isolate was detected by their specific growth and appearance on LIA tubes [19]. Pure colonies of the isolates were tested for citrate utilization ability by stabbing the butt and streaking the slant of Simmons Citrate Agar and incubating at 37°C for 24 hrs [19]. Suspected colonies of* Salmonella* and* Shigella* species were transferred from growth of KIA to nutrient broth with sterile loop and incubated for few hrs. Indole productions were tested by adding 5 drop of Kovac's reagent into the test tubes. Urease production ability of the suspected colony of* Salmonella* and* Shigella* was tested by inoculating heavily over the entire surface of the slant urease medium; the caps were loosened and incubated for an overnight at 37°C [19].

#### 2.5.4. Antibiotics Susceptibility Test

Antibiotics resistance pattern of the isolates was determined using disc diffusion methods [20]. A single pure colony of each isolates was inoculated into the nutrient broth and incubated at 37°C for 24 hrs and their turbidity was adjusted with 0.5 McFarland. The culture was inoculated on Muller Hinton agar using sterilized cotton swab. Discs impregnated with appropriate concentration of antibiotics were placed on inoculated plate. Amoxicillin (30 *μ*g), ceftriaxone (30 *μ*g), chloramphenicol (30 *μ*g), ciprofloxacin (5 *μ*g), gentamicin (10 *μ*g), nalidixic acid (30 *μ*g), and norfloxacin (10 *μ*g) disks were used for the test.* E. coli *(ATCC 25922) was used as reference strain to ensure quality of the data. Zones of inhibition were measured to the nearest mm and results were interpreted according to the CLSI [20].

### 2.6. Data Analysis and Interpretation

The data was edited and analyzed using SPSS version 23. Multivariate logistic regression test, adjusted odds ratio (AOR), and 95% CI (*P* < 0.05 significance level) were used to assess the level of association among prevalence of the pathogens and associated risk factors.

## 3. Results

### 3.1. Sociodemographic and Clinical Characteristics of the Patients

In this study, 422 patients with acute diarrhea and attending Nekemte Referral Hospital from October 2015 to February 2016 were considered. Of these 52.8% were male and the mean age of the study participants was 20.7 years (Table 1). The majority of study participants (83.2%) were urban dwellers and 62.1% of them have the habit of washing their hands regularly after using toilet. Most (83.2%) of the diarrheal patients sources of drinking water was tap whereas 16.6% of them depend on river. Abdominal pain, fever, vomiting, and headache were the clinical symptoms observed among the study population. Abdominal pain was found to be the most common (99.3%) clinical symptom followed by fever and vomiting (Table 1).

### 3.2. Prevalence of* Salmonella *and* Shigella* Species

Out of the total stool specimens, 9.2% were positive for* Salmonella* and* Shigella* species (Table 1). Of these, 7.1% and 2.1% were positive for* Salmonella* and* Shigella *species, respectively. 77.7% of* Shigella* isolates were obtained from male patients. Of all isolates, 46.1% were isolated from patients with age less than 19 years. In contrast, neither* Salmonella* nor* Shigella* species were found in study participants with age of 60 years and above. Our results also indicated that there were no statistically significant associations (*P* > 0.05) between gender, age groups, and prevalence of* Salmonella* and* Shigella *species, whereas poor hand washing habit after toilet (AOR: 4.1, *P*: 0.002), vomiting (AOR: 10.0, *P*: 0.040), and headache (AOR: 4.7, *P*: 0.013) had statistically significant (*P* < 0.05) association with the rate of isolation of the pathogens (data not shown).

### 3.3. Antibiotic Susceptibility Pattern of the Isolates

Of all* Salmonella *isolates, 90% were resistant to amoxicillin (30 *μ*g) whereas only 10%, 6.7%, 6.7%, 3.3%, 3.3%, and 3.3% of the isolates were found to be resistant to gentamicin (10 *μ*g), ciprofloxacin (5 *μ*g), chloramphenicol (30 *μ*g), ceftriaxone (30 *μ*g), norfloxacin (10 *μ*g), and nalidixic acid (30 *μ*g), respectively. Of all, 77.8% of* Shigella* isolates were resistant to amoxicillin, whereas 11.11% of them were found to be resistant to ciprofloxacin, chloramphenicol, gentamicin, and nalidixic acid.* Shigella* isolates were also found to be of high susceptibility to ceftriaxone and norfloxacin followed by ciprofloxacin, nalidixic acid, chloramphenicol, and gentamicin (Table 2). Multiple drug resistances were observed among* Salmonella* and* Shigella* isolates obtained from the participants of this study. Amazingly, one* Salmonella* isolate showed resistance to six antibiotics, while only 10% of the isolates exhibited multidrug resistance to different antibiotics tested in the study. Our results demonstrated that 33.33% of* Shigella* isolates exhibited multidrug resistance to gentamicin and amoxicillin (Table 3).

## 4. Discussion

According to this study, the predominant bacterial pathogens were* Salmonella *(7.11%), indicating that it is common human health problem at the study area. The prevalence of* Salmonella *in this study is in agreement with the result (7.8%) of study done in Bahir Dar [14] and higher than the result of studies done in Gonder (1.08%) [8] and Addis Ababa 3.95% [21], Ethiopia. Similarly, isolation rate of* Salmonella* species obtained in this study is higher than the results of studies in other countries, 3.3% in Nigeria [11] and 1.5% in Turkey [12]. In contrast, our result is lower than studies done in Gonder (17.39%) [22], Harar (11.5%) [13], Butajira (10.5%) [23], and Jimma (10.8%) [16], Ethiopia. The variation could be due to differences in sociodemographic characteristics, geographical location, and study time.

The isolation rate of* Shigella *species obtained in this study is lower than the earlier studies done in Ethiopia; 9.5% in Bahir Dar [14], 9.1% in Addis Ababa [21], 6.9% in Mekelle [15], 6.7% in Harar [13], 4.57% in Gonder [8], 4.5% in Butajira [23], and 29% in Ambo [10]. Comparable results were reported by studies carried out elsewhere 2.9% in Nigeria Abeokuta [11] and 3% in Turkey [12]. In contrast, the isolation rate of* Shigella* species in this study is higher than the results of studies done in Ambo (1.3%) [24] and Jimma (1.1%) [16].

Our results showed that 62.1% of the study participants wash their hands after using latrine. Good hand washing pattern after using latrine has statically significant association with prevalence of* Salmonella* and* Shigella* species. However, there were no statistically significant association between the percentage of patients with abdominal pain, joint pain, and prevalence of the pathogens. But there were statistically significant associations between headache, vomiting, and prevalence of* Salmonella *and* Shigella *species. The infection rate (46.15%) within age group below 19 years might be due to difference in immunity status of the study group.

According to this study, the resistance rate of* Salmonella* was 90% to amoxicillin which is in agreement with reports from other areas of Ethiopia [8, 13, 22, 25]. The possible reason could be due to long time service and wide use of this drug in the country and frequent exposure of* Salmonella* species to it.* Salmonella* species showed that less resistance to gentamicin in this study is lower than result (25%) study done in Gonder [8]. In contrast, the result of this study is higher than results reported from other areas in Ethiopia [13, 21, 23].

On the other hand,* Salmonella* isolates showed lower (3.33%) resistance rate to ceftriaxone and nalidixic acid than results obtained by earlier study done in Hawassa which were 75% and 25%, respectively [25]. Similarly, resistance level of* Salmonella* isolates to chloramphenicol, gentamicin, and nalidixic acid is lower than results of studies conducted in Harar [13], Addis Ababa [21], and Gonder [8]. Resistance rate (3.33%) of* Salmonella* isolates to ceftriaxone observed in this study is slightly higher than earlier studies done in other areas of the country [8, 21, 23]. The proportion (10%) of multidrug-resistant* Salmonella* isolates found in this study is lower than the previous reports [14].

*Shigella* species isolated in this study were highly susceptible to both ceftriaxone and norfloxacin (100%) followed by ciprofloxacin and nalidixic acid. These findings are supported by the results of previous study conducted in other areas of Ethiopia [8, 21, 23]. The resistance rate* Shigella* isolates to amoxicillin was 77.78% which is in agreement with the study done in Gonder (88.2%) [8] and slightly lower than result reported from Addis Ababa (91.4%) [21], Harar (100%) [13], and Hawassa (100%) [25]. On the other hand, low resistant rate of* Shigella* isolates to chloramphenicol and gentamicin (11.11%) observed in this study is contradictory to the results of earlier studies [8, 13, 14, 23, 25] and supported by lower resistance level to gentamicin reported by other studies [11, 14, 21, 23, 26]. More than 33% of* Shigella* isolates were multidrug-resistant which is higher than the previous study [8] in Gonder, Ethiopia.

## 5. Conclusion

Our results revealed 9.2% prevalence of* Salmonella *than* Shigella* species among diarrheal patients in the study area. Antimicrobial susceptibility test results showed that* Salmonella* and* Shigella* species isolated during this study are highly resistant to amoxicillin.* Salmonella* and* Shigella *isolates were highly sensitive to ceftriaxone and norfloxacin. Based on the results of the current study we strongly recommend continuous surveillance on the prevalence and antibiotic resistance pattern of* Salmonella* and* Shigella* species among relevant patients in hospitals and other health centers in the study area which should be basis for empirical therapy. Since* Salmonella *and* Shigella *species were resistant to most common drugs, care should be taken in selecting antimicrobials in treating disease caused by them. Moreover, improving environmental and personal hygiene and providing safe potable water and intensive health education together with more careful use of antimicrobials could conserve antimicrobial efficacy and significantly reduce diarrheal illness.

In this study, the pathogens were not identified to the species level using serological analysis and hence biochemical tests should be substantiated by serological and molecular identification for better taxonomy of the pathogens to species and strain level. This study was conducted only on patients presented with diarrheal case and visited Nekemte Referral Hospital and did not include patients from health centers and private clinics. However, the study verified that further epidemiological study should be conducted on patients attending health centers and private clinics.

## Figures and Tables

**Table 1:** Sociodemographic, clinical characteristics, and prevalence of *Salmonella* and *Shigella* sp. among diarrheal patients attending Nekemte Referral Hospital, October 2015 to February 2016.

Variables	Frequency	Percent (%)	Number of* Salmonella* or *Shigella* species positive patients (%)
Sex			
M	223	52.8	17 (7.6)
F	199	199	22 (11.1)
Age Category (years)			
1–19	225	53.3	18 (8)
19–29	81	19.2	6 (7.4)
30–44	69	16.4	11 (15.9)
45–59	35	8.3	4 (11.4)
>60	12	2.8	0
Educational status			
Illiterate	137	32.5	12 (8.8)
Grade 1–4	70	16.6	4 (5.3)
Grade 5–8	71	16.8	4 (5.6)
Grade 9–12	81	19.2	11 (13.6)
Certificate dip.	57	13.5	7 (12.3)
1st degree & above	6	1.4	1 (16.7)
Hand washing after toilet			
Good	262	62.1	29 (11.1)
Poor	160	37.9	10 (6.3)
Source of drinking water			
Tap	351	83.2	31 (8.8)
River	70	16.6	8 (11.4)
well	1	0.2	0 (0.0)
Residence areas			
Urban	320	91.1	31 (8.8)
Rural	63	8.9	8 (11.3)
Clinical characteristics			
Abdominal pain	419	99.3	38 (9.1)
Vomiting	272	64.5	28 (10.3)
Fever	310	73.5	38 (12.3)
Headache	222	52.6	34 (15.3)
Joint pain	182	43.1	27 (14.8)
Prevalence of *Salmonella* & *Shigella* sp.			
*Shigella *sp.	9	2.1	-
*Salmonella *sp.	30	7.1	-
Total Prevalence	39	9.2	-

*Note*. *Salmonella* and *Shigella* positive values as No (%), sp.: species.

**Table 2:** Antibiotics susceptibility pattern of *Salmonella *and *Shigella *species from diarrheal patients attending Nekemte Referral Hospital, October 2015 to February 2016.

Antimicrobial	*Salmonella *species* N (%)*			*Shigella *species* N (%)*		
	S	I	R	S	I	R
Ceftriaxone	28 (93.33%)	1 (3.33%)	1 (3.33%)	9 (100%)	0 (0%)	0 (0%)
Norfloxacin	29 (96.67%)	0 (0%)	1 (3.33%)	9 (100%)	0 (0%)	0 (0%)
Ciprofloxacin	28 (93.33%)	0 (0%)	2 (6.67%)	8 (88.89%)	1 (11.11%)	0 (0%)
Nalidixic acid	22 (73.33%)	7 (23.33%)	1 (3.33%)	8 (88.89%)	0 (0%)	1 (11.11%)
Chloramphenicol	21 (70%)	7 (23.3%)	2 (6.67%)	7 (77.78%)	1 (11.11%)	1 (11.11%)
Gentamicin	21 (70%)	6 (20%)	3 (10%)	7 (77.78%)	1 (11.11%)	1 (11.11%)
Amoxicillin	0 (0%)	3 (10%)	27 (90%)	2 (22.22%)	0 (0%)	7 (77.78%)

*Parenthesis*. S = sensitive; I = intermediate; R = resistance.

**Table 3:** Multidrug resistance pattern of *Salmonella *and *Shigella *species from diarrheal patients attending Nekemte Referral Hospital, October 2015 to February 2016.

Bacterial Isolates	Number isolate	Antibiotics resisted
*Shigella* species	3 (33.33 %)	GEN, AM

*Salmonella* species	1 (3.33%)	C, AM
	1 (3.33%)	CIP, AM
	1 (3.33%)	C, CIP, CRO, NA, GEN, NOR

*Parenthesis*. GEN = gentamicin; AM = amoxicillin; C = chloramphenicol; CIP = ciprofloxacin; CRO = ceftriaxone; NA = nalidixic acid; NOR = norfloxacin.

## Additional Points

*Availability of Data and Materials*. Data supporting this research article are available from the corresponding author or first author on reasonable request.
